# Eplerenone Attenuates Fibrosis in the Contralateral Kidney of UUO Rats by Preventing Macrophage-to-Myofibroblast Transition

**DOI:** 10.3389/fphar.2021.620433

**Published:** 2021-02-24

**Authors:** Yunzhao Xiong, Yi Chang, Juan Hao, Cuijuan Zhang, Fan Yang, Zheng Wang, Yunmeng Liu, Xiangting Wang, Shengyu Mu, Qingyou Xu

**Affiliations:** ^1^ Graduate School, Hebei University of Chinese Medicine, Shijiazhuang, China; ^2^ Hebei Key Laboratory of Integrative Medicine on Liver-Kidney Patterns, Hebei University of Chinese Medicine, Shijiazhuang, China; ^3^ Department of Pharmacology and Toxicology, University of Arkansas for Medical Sciences, Little Rock, AR, United States; ^4^ Department of Internal Medicine, Hebei University of Chinese Medicine, Shijiazhuang, China

**Keywords:** UUO contralateral kidney, renal fibrosis, aldosterone, MR blockers, macrophage-to-myofibroblast-transition (MMT)

## Abstract

Severe renal fibrosis often occurs in obstructive kidney disease, not only in the obstructed kidney but also in the contralateral kidney, causing renal dysfunction. Although the mechanisms of injury in obstructed kidney have been studied for years, the pathogenesis of fibrosis in the contralateral kidney remains largely unknown. Here, we examined long-term unilateral ureteral obstruction (UUO) model in male Sprague–Dawley rats and found that macrophage-to-myofibroblast transition (MMT) is contributing to renal fibrosis in the contralateral kidney of UUO rats. Interestingly, this process was attenuated by treatment of eplerenone, a specific blocker of the mineralocorticoid receptor (MR). *In-vitro*, stimulating MR in primary cultured or cell line macrophages enhances MMT, which were also inhibited by MR blockade. Collectively, these findings provide a plausible mechanism for UUO-induced injury in the contralateral kidney, suggesting the benefit of using MR blockage as a part of treatment to UUO to protect the contralateral kidney thereby preserve renal function.

## Introduction

Chronic kidney disease (CKD) is increasingly recognized as a global public health problem, since both the incidence and prevalence rates of CKD are increasing worldwide, particularly among aging population, resulting in morbidity, mortality, and high medical costs (Xie et al., 2018). One of the major causes of CKD is obstructive nephropathy, which leads to acute injury in the obstructed kidney and chronic fibrosis in both obstructed and contralateral kidneys, thereby progressively decreasing renal function, resulting in renal failure (Nishida and Hamaoka, 2008; Romagnani et al., 2017). In the past decades, numerous previous studies have revealed the mechanisms of acute injury in the obstructed kidney. However, the pathogenesis of chronic fibrosis in the contralateral kidney remains to be investigated. It is well accepted that myofibroblasts are an important source of collagen production during renal fibrosis (Nishida and Hamaoka, 2008; Ricardo et al., 2008), however, the cell origin of these myofibroblasts remains unclear. Renal interstitial fibrosis often occurs after inflammation and invasive immune reaction (Ricardo et al., 2008). In recent years, accumulating evidence indicate that in rodent models of renal injury or in patients with progressive CKD, a proportion of myofibroblasts in their injured kidneys may from bone marrow-derived monocytes/macrophages, identified by co-expression of macrophage makers (CD68 or F4/80) and myofibroblast marker α-smooth muscle actin (α-SMA) in conjunction with producing collagens (Yang et al., 2013; Nikolic-Paterson et al., 2014; Wang et al., 2016). However, whether this macrophage-to-myofibroblast transition (MMT) also occurs in the healthy contralateral kidney in obstructive nephropathy and by what mechanism are important questions that remain to be answered.

We and others have previously reported renal protective effect of eplerenone, a mineralocorticoid receptor (MR) blocker, in various rodent models of renal injury, including UUO (Furumatsu et al., 2008; Kuriyama et al., 2009; Toto, 2010). Especially, our group has recently reported elevated plasma levels of aldosterone (Ald), a MR specific agonist, in UUO animals (Wang, C. H. et al., 2017). These observations led us to further speculate that in UUO, the activation of MR by elevated aldosterone in plasma not only participates in renal injury and fibrosis in the obstructed kidney, but also contributes to the fibrosis in the contralateral kidney, exacerbating CKD and renal failure. Moreover, we suspect that MR activation may be involved in the mechanism of MMT in the chronic contralateral kidney injury in UUO. Hence, blockage of MR may provide therapeutic effects to the chronic fibrosis of the contralateral kidney in obstructive kidney disease.

In the current study, we will examine chronic fibrosis in the contralateral kidney of UUO rats, to determine whether activation of MR in the contralateral kidney participating in the mechanism of MMT, which mediates interstitial fibrosis, and eventually leading to CKD and renal failure.

## Results

### MR Blockade by Eplerenone Protects Contralateral Kidney From UUO-Induced Chronic Fibrosis

**FIGURE 1:**
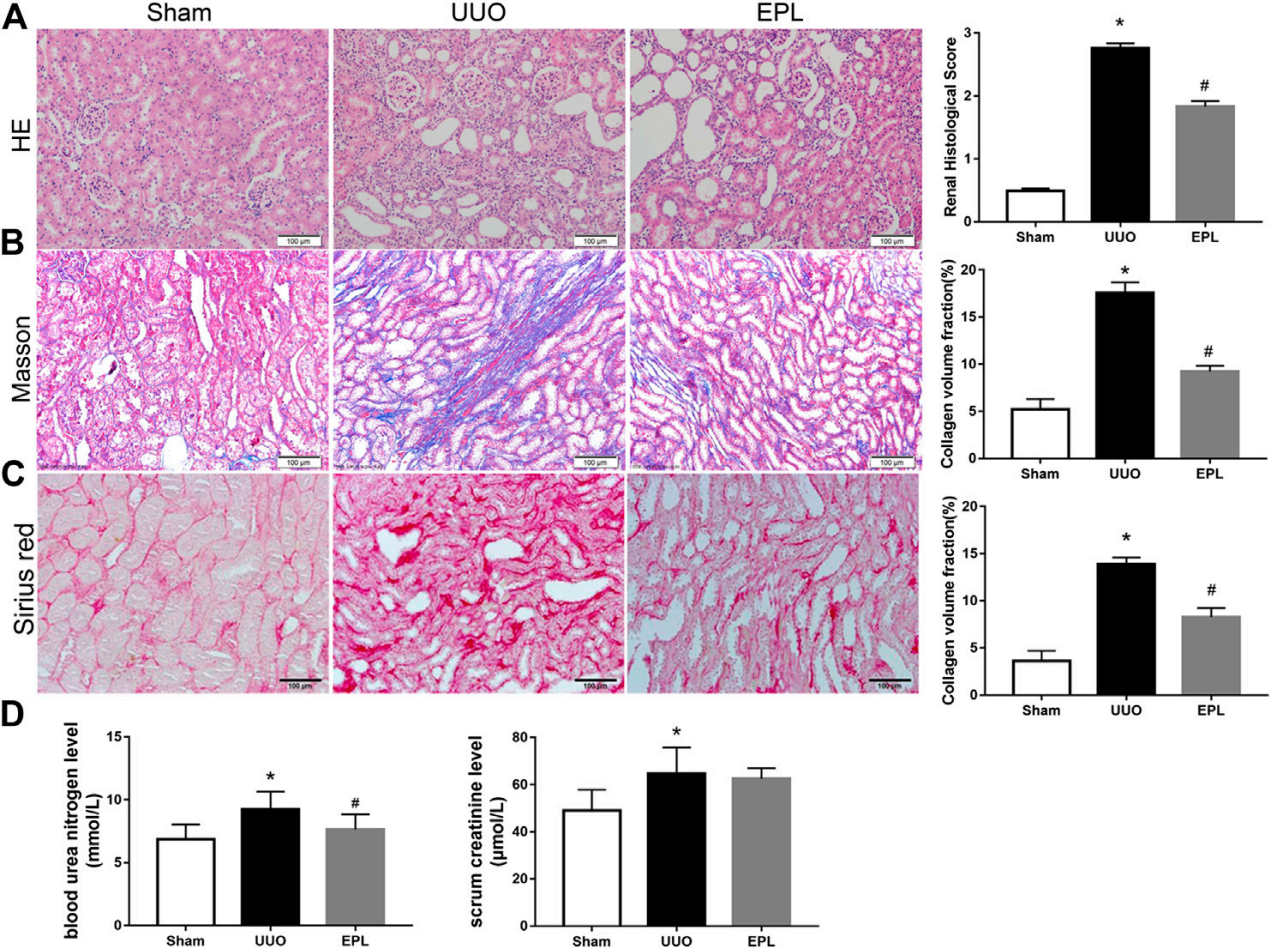
Effects of long term-UUO and treatment of eplerenone (EPL) on renal histology, fibrosis, and function of the contralateral kidneys. Kidney sections from all groups were stained with HE for morphology changing **(A)**, Masson's Trichrome (Masson) for fibrosis **(B)**, and Sirius red for collagen deposition **(C)**. Images are representative for *n* = 12 rats each group. **(D)** BUN and Scr in serum were evaluated for renal function. Renal scores and quantification data are presented as mean ± SEM, *n* = 6. *p < 0.05 vs. the sham, #*p* < 0.05 vs. the UUO.

UUO is a classic model in rodents to study obstructive kidney disease and renal fibrosis (Suman et al., 2016). UUO leads to acute kidney injury in the obstructed kidney rapidly, but in the chronic phase, both obstructed kidney and contralateral kidney exhibit fibrosis leading to progressive CKD. The target of this study focuses on chronic injury in the contralateral kidneys after obstruction; therefore, we harvested contralateral kidneys from UUO rats 180 days after they received UUO surgery. HE staining for renal morphology confirmed that long term-UUO induces injury in the contralateral kidney, by observing dilated distal tubules and cast formation (Figure 1A). Moreover, Masson staining showed significantly higher collagen deposition and fibrosis in the contralateral kidneys of long term-UUO rats compared to sham kidneys (Figure 1B). Similar results also observed by using Sirius red staining for collagens in these kidneys (Figure 1C). Consistent with pathology observations, renal function tests for blood urea nitrogen and serum creatinine level (Figure 1D) also revealed impaired renal function in long term-UUO rats compared with sham rats. It is noteworthy that the treatment of eplerenone (EPL) to these long term-UUO rats protected their contralateral kidneys from chronic fibrosis (Figures 1B,C), even though some cast formation were still observed in the HE stained kidney sections (Figure 1A). These findings suggest that long term-UUO induces severe fibrosis and chronic injuries in the contralateral kidneys, thereby impairing renal function. Interestingly blocking MR by EPL attenuated massive fibrosis in the contralateral kidneys of UUO rats, indicating that MR activation may be involved in the pathogenesis of developing chronic renal fibrosis in the contralateral kidneys of UUO rats.

### Eplerenone Attenuated UUO-Induced MMT in the Contralateral Kidney

**FIGURE 2:**
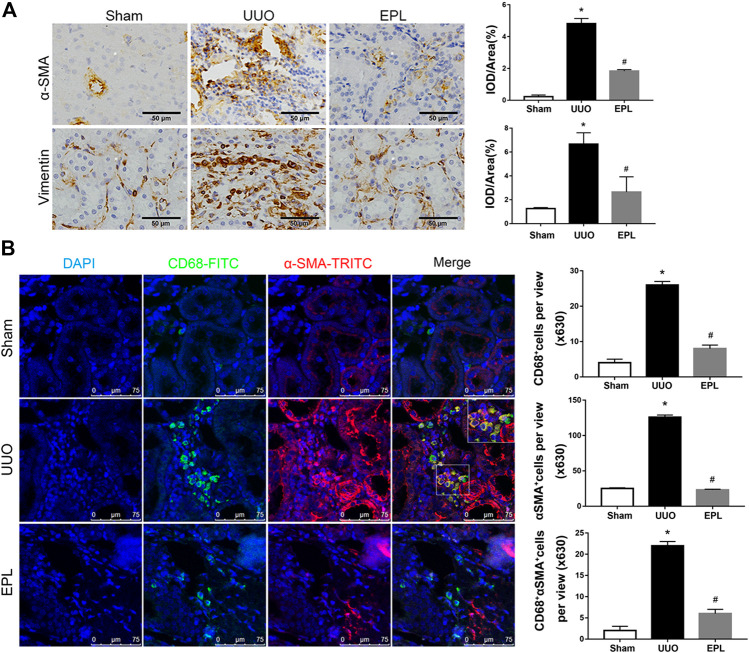
Macrophage-to-myofibroblast transition (MMT) in the contralateral kidneys of long term-UUO rats. **(A)** Immunohistochemistry staining using antibodies against α-SMA **(upper panel)** and Vimentin **(lower panel)** to examine renal infiltration of myofibroblasts in the kidneys. **(B)** immunofluorescent multi-staining of kidney sections with antibodies against macrophage marker CD68 (FITC, green), and myofibroblast marker α-SMA (TRITC, red) for identifying MMT (cells co-expressing both markers indicates MMT, nuclei were stained with DAPI in blue). Images are representative for *n* = 12 rats each group. Staining quantification data are presented as mean ± SEM, *n* = 6. *p < 0.05 vs. the sham, #*p* < 0.05 vs. the UUO.

Renal fibrosis is a leading factor resulting in kidney destruction and loss of renal function. The activation of α-smooth muscle actin (α-SMA) positive myofibroblasts plays a key role in this process (Eddy, 2013; Klingberg et al., 2013). To examine the role of myofibroblasts in chronic injury of the contralateral kidney of long-term UUO rats, we stained these kidneys with antibodies against α-SMA and vimentin, specific markers used to identify myofibroblasts (Eddy, 2013; Klingberg et al., 2013; Bagalad et al., 2017). We found massive infiltration of myofibroblasts in the contralateral kidneys of rat exposed to long-term UUO (Figure 2A), and these effects also were prevented by treatment of EPL (Figure 2A).

Monocytes/macrophages are highly involved in the process of renal injury, repair and fibrosis. One significant feature in kidney biopsy specimens of patients with CKD is the presence of massive macrophage infiltration and severe fibrosis (Wang, Y. Y. et al., 2017). To determine whether macrophages are involved in UUO-induced renal fibrosis in the contralateral kidney of UUO rats, we performed immunofluorescent co-staining using specific antibodies against monocyte/macrophage marker CD68 and myofibroblast marker α-SMA. As shown in Figure 2B, massive infiltration of CD68^+^ macrophages were observed in the contralateral kidneys of UUO rats compared to sham rats. It is worthy to note that most of these renal infiltrated macrophages in the contralateral kidneys of UUO rats co-expressing α-SMA, indicating macrophage-to-myofibroblast transition (MMT), and the co-staining of CD68 and α-SMA in the UUO-contralateral kidneys were largely diminished by the treatment of eplerenone (Figure 2). These data suggest that MMT is contributing to renal fibrosis and pathology in the contralateral kidneys of long term-UUO rats.

**FIGURE 3:**
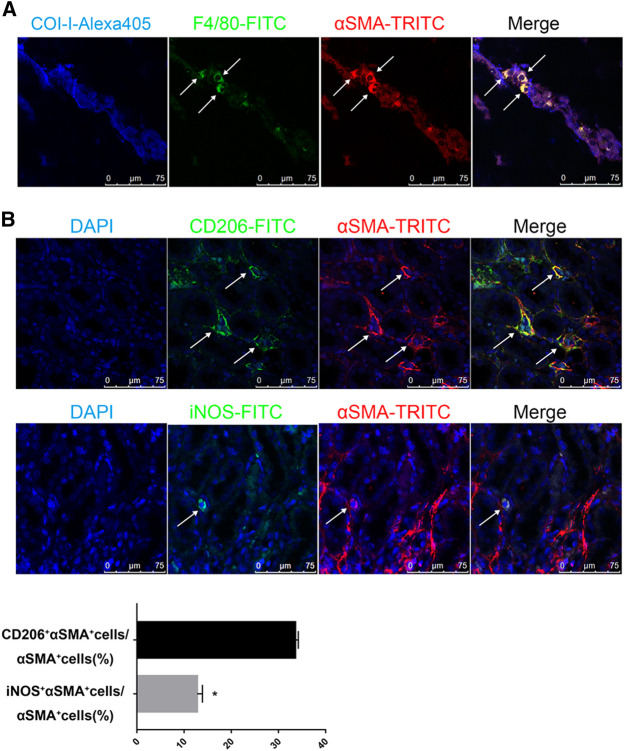
M2 macrophages are the major source of MMT in the contralateral kidneys of long term-UUO rats. **(A)** immunofluorescent multi-staining of kidney sections with antibodies against collagen I (blue), macrophage marker F4/80 (green), and α-SMA (red). **(B)** fluorescent co-staining of α-SMA (red) with M2 macrophage marker CD206 (green, upper panel) or M1 macrophage marker iNOS (green, lower panel), nuclei were stained with DAPI in blue. Images are representative for *n* = 12 rats each group. Quantification data are presented as mean ± SEM, *n* = 6. *p < 0.05 vs. the sham, #*p* < 0.05 vs. the UUO.

Next, we stained the UUO-contralateral kidneys with multi-antibodies against Collagen-I, F4/80 (macrophage specific marker) and α-SMA. As expected, cells co-expressing F4/80 and α-SMA were surrounded by collagen-I, suggesting production of collagen-I from MMT cells (Figure 3A). Moreover, we explored subtypes of macrophages contributing to MMT by co-staining α-SMA with macrophage subfamily markers CD206 (M2 macrophages) or iNOS (M1 macrophages). Results further revealed that M2 macrophages are the major contributor to MMT in the contralateral kidneys of long term-UUO rats (Figure 3B), which is consistent with previous acute study using UUO kidneys (Wang, Y. Y. et al., 2017).

Taken together, data from Figures 2, 3 suggest a potential mechanism for the pathogenesis of fibrosis in the contralateral kidney of UUO rats, that at least a great portion of myofibroblasts developed by MMT from renal infiltrated macrophages, particularly M2 macrophages, contributing to collagen production and subsequently developing renal fibrosis. Moreover, these effects are inhibited by eplerenone, a MR specific blocker, leading us to speculate an important role for MR involved in the process of MMT and thereafter renal fibrosis.

### Activation of MR Stimulate Macrophage-to-Myofibroblast Transition (MMT) in Macrophages

**FIGURE 4:**
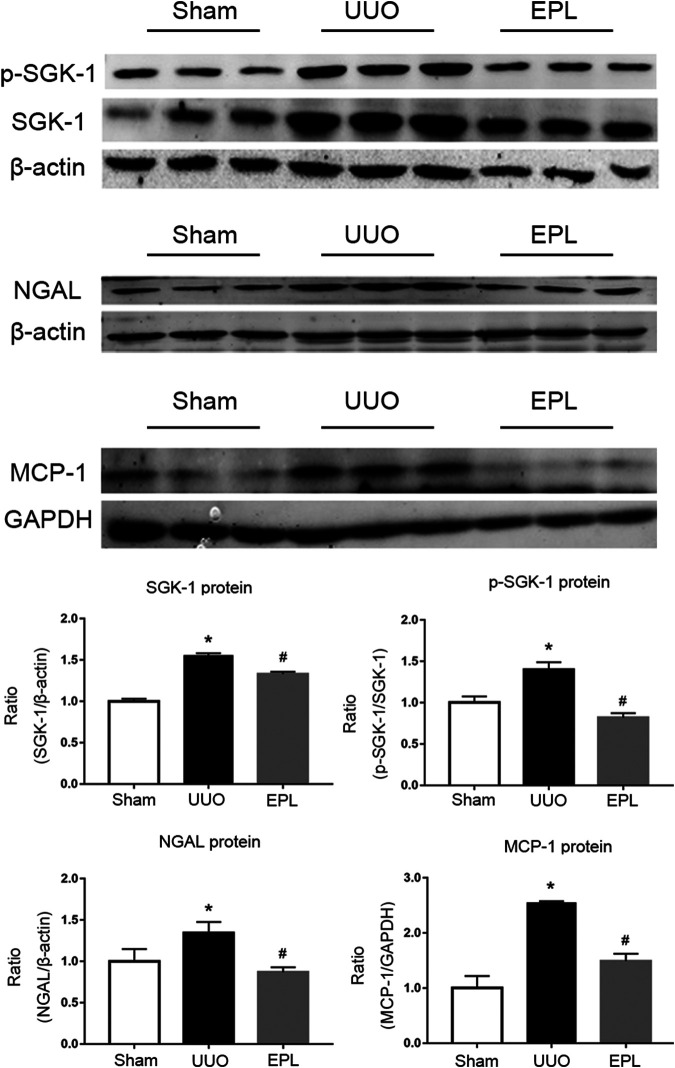
Effects of long term-UUO and treatment of eplerenone (EPL) on the expression of MR downstream molecules. Western blot for protein level expression of SGK1, (p)SGK1, NGAL and MCP-1 in the contralateral kidneys of rats treated with Sham, UUO and UUO + eplerenone (EPL). Data are mean ± SEM for *n* = 12 rats each group. *p < 0.05 vs. the sham, #*p* < 0.05 vs. the UUO.

Plasma aldosterone level is elevated in long term-UUO rats, which may lead to higher activity of MR (Wang, Y. Y. et al., 2017). In the current study, we further confirmed activation of MR in the contralateral kidney of UUO rats by detecting molecules downstream of MR using western blots. Consistent with our previous findings (Wang, C. H. et al., 2017), we found greater expression of SGK-1, phosphorylated-SGK-1, NGAL and MCP-1 in the contralateral kidneys of UUO rats compared to the contralateral kidneys from Sham rats (Figure 4), indicating higher MR activity in these kidneys, and these effects were inhibited by MR blocker eplerenone (Figure 4), as we expected.

**FIGURE 5:**
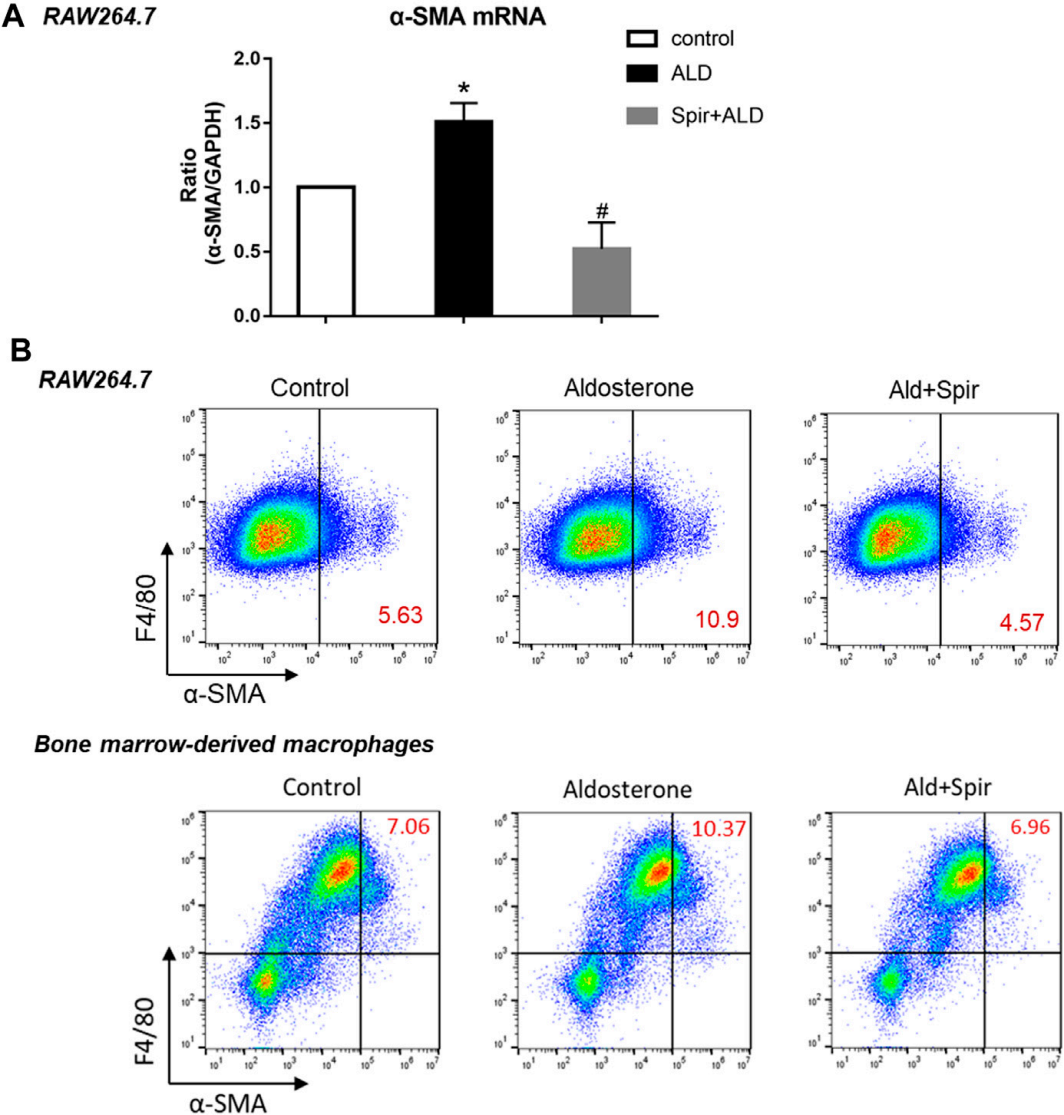
Effects of aldosterone and MR blocker spironolactone (Spir) on the expression of α-SMA in macrophages. **(A)** mRNA levels expression of α-SMA in cell line macrophages, RAW264.7 cells, treated with or without additional aldosterone and Spir. Data are mean ± SEM. *n* = 3 independent tests. *p < 0.05 vs. control; #*p* < 0.05 vs. Aldosterone. **(B)** Two-color flow cytometry analysis show protein level expression of α-SMA in macrophage cell line (RAW264.7, upper panel) and mouse bone marrow derived macrophages **(lower panel)**. Numbers in red color indicating the percentage of MMT cells with α-SMA^+^, F4/80^+^. Data are representative for *n* = 5–7 independent tests.

To further investigate the role of MR activation in MMT, we tested the effects of additional aldosterone and MR blockade on the expression of α-SMA in macrophages. Addition of aldosterone stimulated mRNA expression of α-SMA in macrophage cell line RAW 264.7 cells, and this effect was blocked by specific MR inhibitor spironolactone (Figure 5A, detected by Taqman realtime PCR). Consistent with realtime-PCR results, aldosterone stimulation also upregulated protein level expression of α-SMA in RAW 264.7 cells, and this upregulation was attenuated by MR blocker spironolactone as well (Figure 5B, upper panel, detected by flow cytometer). As expected, similar data was also observed in mouse bone marrow-derived macrophages (Figure 5B, lower panel, F4/80^+^ cells, detected by flow cytometer), confirmed above results using cell line. The stimulating effects of aldosterone on the expression of α-SMA in macrophages were clearly blocked by MR specific blocker spironolactone, suggesting activation of MR contributes to MMT in macrophages.

Taken together, our results suggest that in the chronic phase of obstructive kidney disease, activated MR stimulate MMT for renal infiltrated macrophages to transdifferentiate into myofibroblasts, producing excessive collagen, exacerbating renal fibrosis in the contralateral kidney. Moreover, MR blockers diminish MMT and protect the contralateral kidney from fibrosis and further chronic renal injury.

## Discussion

Obstructive kidney disease is one of the major causes of renal injury, which may lead to renal failure (Tseng and Stoller, 2009). UUO, as a classic preclinical model of obstructive kidney disease have been studied for decades. However, the majority of those studies focused on the acute kidney injury of the obstructed kidneys, and the mechanisms causing chronic injury to the non-obstructed contralateral kidneys remain largely unknown. In recent years, a growing body of evidence has shown that obstructive kidney injury-induced CKD or renal failure are related to both the obstructed kidney and the non-obstructed contralateral kidney (Wang, C. H. et al., 2017; Ma et al., 2019). Thus, we focus on investigating the mechanisms of inflammation and fibrosis in the contralateral kidneys of UUO animals, hopefully to improve therapeutic strategies to help gain better outcomes for patients suffering obstructive kidney disease.

Previous studies from our laboratory have reported observations of higher level plasma aldosterone in UUO rats compared with sham rats. Moreover, the activation of MR contributes to chronic inflammation in the contralateral kidneys of UUO rats compared to sham rats (Wang, C. H. et al., 2017; Ma et al., 2019). In the current study, we further determined the contributing role of MR-activation in the pathogenesis of renal fibrosis in the contralateral kidneys of UUO rats, enhancing MMT in the kidney, promoting renal infiltrated macrophages to transdifferentiate into myofibroblasts.

In this study, our first novel finding is that the MR blocker eplerenone efficiently prevented chronic development of fibrosis in the contralateral kidney of UUO animals, and thereby protected the contralateral kidney from further damage. MR has been reported to contribute to fibrosis in many organs (Shrestha et al., 2019); however, its role in participating in fibrosis in the contralateral kidney in UUO rats has not previously been revealed. Here, we found the mechanism of these effects is at least in part, by reducing accumulation of myofibroblasts in the kidney. Macrophage to myofibroblast transition (MMT) is one of the important mechanisms for the origin of myofibroblasts in solid organs (Wynn and Barron, 2010; Klingberg et al., 2013; Emily et al., 2015; Kryczka and Boncela, 2015; Kurose and Mangmool, 2016). By co-staining macrophage marker and a maker for myofibroblasts, our data further elucidated that attenuating MMT is an important mechanism for eplerenone to prevent accumulation of myofibroblasts in the contralateral kidneys of UUO rats.

Our second novel finding in this study is that MR also directly participates in MMT of macrophages. MR blockade is a well-known antihypertensive treatment, and high blood pressure often occurs in obstructive kidney diseases (El-Dahr et al., 1993; Al-Marhoon et al., 2015). Although our *in vivo* data cannot exclude the possible role of BP in MMT, our *in vitro* studies using cultured cells without BP effects suggest aldosterone-MR-induced MMT is not BP dependent. Our data in Figure 5 demonstrated that Aldosterone stimulates macrophages to express higher level of α-SMA, a marker for myofibroblasts, indicating enhanced MMT, and these effects are significantly blocked by specific MR blocker spironolactone. Similar results were observed in both the non-polarized macrophage cell line R264.7 cells and well differentiated mouse bone-marrow-derived macrophages.

One limitation in this study is that we do not exactly know the molecular mechanism by which activated MR participates in macrophage-differentiation. One of the possible mechanisms for reducing MMT in the contralateral kidney of UUO rats is that the treatment with eplerenone decreased renal infiltration or accumulation of macrophages. Indeed in our immunostaining sections, we observed higher macrophage accumulation in the contralateral kidneys of UUO rats (Figure 2). This is consistent with our previous report that contralateral kidneys of UUO rats demonstrate higher level of chronic inflammation (Wang, C. H. et al., 2017). However, other than eliminating the number of macrophages in the kidney, whether MR is otherwise involved in the MMT process remains to be tested. Using both *in vivo* and *in vitro* studies, we determined that MR blockers also attenuated MMT in macrophages as one of the mechanisms involved in reducing collagen deposition and renal fibrosis in the contralateral kidney of UUO rats. Therefore, future study will be needed to further address the precise molecular mechanisms of MR stimulating MTT. In summary, our study indicated an important benefit of giving MR blocker to subjects with obstructive kidney disease, for protecting the contralateral kidney from chronic injury and fibrosis. Moreover, we suggest the mechanisms of this protection are probably by preventing both macrophage-infiltrations in the contralateral kidney and the MMT process of macrophages.

## Materials and Methods

### Animals and In-Vivo Experimental Models

Male Sprague–Dawley (SD) rats (about 7 weeks old, body weight 200 ± 10 g) were purchased from Hebei Medical University Animal Center and used for this study. The rats were maintained with standard rat chow and tap water at room temperature under a 12-h light/12-h dark cycle. Animal care followed the criteria of the Ethics Committee on Animal Experimentation of Hebei Medical University. 36 SD rats were randomly assigned to sham group, UUO group and UUO with eplerenone treatment group (UUO + Epl, *n* = 12 each). UUO and sham surgeries were performed as described previously (Truong et al., 2011). After surgeries, eplerenone (Pfizer, United States) was given to UUO + Epl group via diet at the dose of 1.25 g/kg diet (equal to 100 mg/kg/day) for 6 months and other groups of rats were fed on regular chow. 180 days after UUO, all animals were sacrificed and the kidneys on the contralateral side of UUO or sham surgeries were collected for histological and protein analysis. Serum sample from all rats were used to test renal function using BUN and Scr screening kits from Beckman Coulter.

### Histological Analysis, Immunohistochemistry and Immunofluorescence

The contralateral kidneys were dehydrated with alcohol, embedded in paraffin blocks after fixation overnight in 4% paraformaldehyde (PFA). Paraffin blocks were cut into 5 μm sections for HE, Masson, Sirius red staining and immunohistochemistry for α-smooth muscle actin (α-SMA; Abcam) and vimentin (Abcam). Light images were observed and pictured using a Leica BX53 optical microscope.

For fluorescent stainings, kidneys were irrigated by 4%PFA, dehydrated in 30% sucrose solution and frozen in OCT compound. 7 μm kidney sections were cut using freezing microtome, and prepared for staining with the following fluorescent-conjugated antibodies: Alexa Fluor 555-conjugated alpha smooth muscle Actin (Abcam), FITC-conjugated F4/80 (EterLife) or unconjugated antibodies anti-collagen-I (Col-I), anti-CD68, anti-iNOS and antiCD206 (all from Abcam) flowed by fluorescent secondary staining. After staining, sections were incubated with DAPI for nucleus staining and sealed for photography using a Leica SP8 confocal microscope.

HE staining sections were examined by two nephrologists and scored from 0 to 3 (normal to severe). Other image quantifications were performed using Image J or Image-Pro Plus 6.0 software.

### Protein Extraction and Western Blot Analysis

The contralateral kidneys were homogenized in RIPA buffer for protein extraction. Western blots employed SDS-PAGE and PVDF membranes. After blocking nonspecific bindings with 5% nonfat milk, the membranes were incubated with primary antibodies against SGK-1, p-SGK-1, MCP-1 (all from Abcam), and NGAL (LCN2, ABclonal) at 1:500–1:1,000 dilution overnight at 4°C. Next day, Blots were then incubated with fluorescein-conjugated secondary antibodies for 1 h at room temperature, and scanned with Odyssey Infrared Imaging System (LI-COR, Lincoln, NE, United States). GAPDH and β-actin antibodies were used for normalization of protein loading.

### Reverse Transcription and Quantitative Real-Time PCR

Total RNA was isolated using RNeasy plus mini kit (Qiagen) following the protocol recommended by the manufacturer. Superscript III kit (Thermo Fisher) was used for reverse transcription of RNA, and realtime PCR was performed using Taqman reaction system (Thermo Fisher) on an Mx3005p realtime PCR instrument. GAPDH was used as a housekeeping transcript for normalization, and relative realtime PCR result were calculated using the 2^–ΔΔCT^ method.

### Cell Culture Treatment and Flow Cytometry

Raw264.7 macrophages and bone marrow differentiated monocyte/macrophages were maintained in culture media with 10% heat-inactivated FBS at 37°C in an incubator with a humidified atmosphere and 5% CO_2_. 10^–7^ M aldosterone (Ald) was used for Ald treatment and in Ald + Spir group cells were pre-treated with 10 uM spirolactone 1 h prior to Ald treatment. 24 h after the treatment, all cells were harvested and stained with PE-conjugated anti-moue F4/80 (Biolegend), APC-conjugated anti-α-SMA (Abcam) for 1 h in dark tubes. Cells were analyzed in a BD Accuri C6 flow cytometer, viable singlet cells were selected by FSC/SSC gating, and data were further analyzed using FlowJo 10 software.

### Mouse Bone Marrow-Derived Macrophages

Full protocol of mouse bone marrow-derived macrophages was obtained from Dr. Tiffany Weinkopff in University of Arkansas for Medical Sciences. Briefly, fresh bone marrow (BM) cells were harvested from mice in cRPMI media and cultured in MCSF conditioned macrophage media for 7 days (fresh media added at day 3). After days 7, cells were harvested and used for experiments. Flow cytometry using F4/80 antibody confirmed most cells used for experiments are macrophages.

### Statistical Analysis

All data were expressed as mean ± SEM. The statistical comparisons between groups were determined using student *t*-test for two groups or one-way ANOVA followed by Tukey’s post hoc tests for multi-groups, with *p* value < 0.05 being considered statistically significant.

## Data Availability

The original contributions presented in the study are included in the article/Supplementary Material, further inquiries can be directed to the corresponding authors.

## References

[B1] Al-Marhoon M. S. Bayoumi R. Al-Farsi Y. Al-Hinai A. Al-Maskary S. Venkiteswaran K. (2015). Urinary stone composition in Oman: with high incidence of cystinuria. Urolithiasis 43, 207–211. 10.1007/s00240-015-0763-7 25805105

[B2] Bagalad B. S. Mohan Kumar K. P. Puneeth H. K. (2017). Myofibroblasts: master of disguise. J. Oral Maxillofac. Pathol. 21 (3), 462–463. 10.4103/jomfp.JOMFP_146_15 PMC5763885 29391737

[B3] Eddy A. A. (2013). The origin of scar-forming kidney myofibroblasts. Nat. Med. 19 (8), 964–966. 10.1038/nm.3299 23921738

[B4] El-Dahr S. S. Gee J. Dipp S. Hanss B. G. Vari R. C. Chao J. (1993). Upregulation of renin-angiotensin system and downregulation of kallikrein in obstructive nephropathy. Am. J. Physiol. 264 (5), 874–881. 10.1152/ajprenal.1993.264.5.F874 8498541

[B5] Emily H. Rachel B. Fallon P. G. (2015). Macrophage and innate lymphoid cell interplay in the genesis of fibrosis. Front. Immunol. 6 (10), 597. 10.3389/fimmu.2015.00597 26635811 PMC4655423

[B6] Furumatsu Y. Nagasawa Y. Tomida K. Mikami S. Kaneko T. Okada N. (2008). Effect of renin-angiotensin-aldosterone system triple blockade on non-diabetic renal disease: addition of an aldosterone blocker, spironolactone, to combination treatment with an angiotensin-converting enzyme inhibitor and angiotensin II receptor blocker. Hypertens. Res. 31 (1), 59–67. 10.1291/hypres.31.59 18360019

[B7] Klingberg F. Hinz B. White E. S. (2013). The myofibroblast matrix: implications for tissue repair and fibrosis. J. Pathol. 229 (2), 298–309. 10.1002/path.4104 22996908 PMC4005341

[B8] Kryczka J. Boncela J. (2015). Leukocytes: the double-edged sword in fibrosis. Mediat. Inflamm. 2015 (4), 1–10. 10.1155/2015/652035 PMC4629055 26568664

[B9] Kuriyama S. Sugano N. Ueda H. Otsuka Y. Kanzaki G. Hosoya T. (2009). Successful effect of triple blockade of renin-angiotensin-aldosterone system on massive proteinuria in a patient with chronic kidney disease. Clin. Exp. Nephrol. 13 (6), 663–666. 10.1007/s10157-009-0213-3 19629623

[B10] Kurose H. Mangmool S. (2016). Myofibroblasts and inflammatory cells as players of cardiac fibrosis. Arch. Pharm. Res. 39 (8), 1100–1113. 10.1007/s12272-016-0809-6 27515051

[B11] Ma X. Chang Y. Xiong Y. Wang Z. Wang X. Xu Q. (2019). Eplerenone ameliorates cell pyroptosis in contralateral kidneys of rats with unilateral ureteral obstruction. Nephron 142 (3), 233–242. 10.1159/000497489 30799394

[B12] Nikolic-Paterson D. J. Wang S. Lan H. Y. (2014). Macrophages promote renal fibrosis through direct and indirect mechanisms. Kidney Int. Suppl. 4 (1), 34–38. 10.1038/kisup.2014.7 PMC4536961 26312148

[B13] Nishida M. Hamaoka K. (2008). Macrophage phenotype and renal fibrosis in obstructive nephropathy. Nephron Exp. Nephrol. 110 (1), 31–36. 10.1159/000151561 18724069

[B14] Ricardo S. D. Van Goor H. Eddy A. A. (2008). Macrophage diversity in renal injury and repair. J. Clin. Invest. 118 (11), 3522–3530. 10.1172/JCI36150 18982158 PMC2575702

[B15] Romagnani P. Remuzzi G. Glassock R. Levin A. Jager K. J. Tonelli M. (2017). Chronic kidney disease. Nat. Rev. Dis. Primers. 3, 17088. 10.1038/nrdp.2017.88 29168475

[B16] Shrestha A. Che R. C. Zhang A. H. (2019). Renal fibrosis: mechanisms and therapies. Singapore: Springer Press.

[B17] Suman R. Alexander D. Moshe L. Seth F. Enrico G. (2016). Characterizing fibrosis in UUO mice model using multiparametric analysis of phasor distribution from FLIM images. Biomed. Opt. Express. 7 (9), 3519–3530. 10.1364/BOE.7.003519 27699117 PMC5030029

[B18] Toto R. D. (2010). Aldosterone blockade in chronic kidney disease: can it improve outcome? Curr. Opin. Nephrol. Hypertens. 19 (5), 444–449. 10.1097/MNH.0b013e32833ce6d5 20625290 PMC5691605

[B19] Truong L. D. Gaber L. Eknoyan G. (2011). Obstructive uropathy. Contrib. Nephrol. 169, 311–326. 10.1159/000314578 21252529

[B20] Tseng T. Y. Stoller M. L. (2009). Obstructive uropathy. Clin. Geriatr. Med. 25 (3), 437–443. 10.1016/j.cger.2009.06.003 19765491

[B21] Wang C. H. Wang Z. Liang L. J. Wang X. T. Ma X. L. Liu B. B. (2017). The inhibitory effect of eplerenone on cell proliferation in the contralateral kidneys of rats with unilateral ureteral obstruction. Nephron 136 (4), 328–338. 10.1159/000473702 28402979

[B22] Wang S. Meng X. M. Ng Y. Y. Ma F. Y. Zhou S. Zhang Y. (2016). TGF-beta/Smad3 signalling regulates the transition of bone marrow-derived macrophages into myofibroblasts during tissue fibrosis. Oncotarget 7 (8), 8809–8822. 10.18632/oncotarget.6604 26684242 PMC4891006

[B23] Wang Y. Y. Jiang H. Pan J. Huang Y. C. Cheng Y. Feng H. (2017). Macrophage-to-myofibroblast transition contributes to interstitial fibrosis in chronic renal allograft injury. J. Am. Soc. Nephrol. 28 (7), 2053–2067. 10.1681/ASN.2016050573 28209809 PMC5491278

[B24] Wynn T. A. Barron L. (2010). Macrophages: master regulators of inflammation and fibrosis. Semin. Liver Dis. 30 (03), 245–257. 10.1055/s-0030-1255354 20665377 PMC2924662

[B25] Xie Y. Bowe B. Mokdad A. H. Xian H. Yan Y. Li T. (2018). Analysis of the Global Burden of Disease study highlights the global, regional, and national trends of chronic kidney disease epidemiology from 1990 to 2016. Kidney Int. 94 (3), 567–581. 10.1016/j.kint.2018.04.011 30078514

[B26] Yang J. Lin S. C. Chen G. He L. Hu Z. Chan L. (2013). Adiponectin promotes monocyte-to-fibroblast transition in renal fibrosis. J. Am. Soc. Nephrol. 24 (10), 1644–1659. 10.1681/ASN.2013030217 23833260 PMC3785282

